# Bridging Old and New in Pain Medicine: An Historical Review

**DOI:** 10.7759/cureus.43639

**Published:** 2023-08-17

**Authors:** Antonella Paladini, Jose Barrientos Penaloza, Ricardo Plancarte Sanchez, Tolga Ergönenç, Giustino Varrassi

**Affiliations:** 1 Life, Health & Environmental Sciences (MESVA), University of L'Aquila, L'Aquila, ITA; 2 Surgery, Hospital Materno Infantil, La Paz, BOL; 3 Anesthesiology, University Autonomous of Mexico, Mexico City, MEX; 4 Anesthesia and Reanimation, Morphological Madrid Research Center, Madrid, ESP; 5 Anesthesia and Reanimation, Akyazi Hospital Pain and Palliative Care, Sakarya, TUR; 6 Pain Medicine, Paolo Procacci Foundation, Rome, ITA

**Keywords:** hypodermic needles, gate control theory of pain, opioids, history of pain medicine, pain medicine

## Abstract

Pain is both one of the oldest complaints known to medicine and a field for some of medicine’s latest breakthroughs and innovations. Pharmacologic treatment of pain is one of the oldest remedies, and opioids have been used since ancient times as an effective pain reliever but with certain specific risks for abuse. Greater knowledge of opioids led to a more thorough understanding of the complexities of pain, which may have any number of mechanisms. A greater understanding of nerve fibers and pain signaling led to the development of more drugs and the more targeted delivery of analgesics using the hollow needle. The hollow needle changed pain treatment and led to percutaneous injections and what would later become interventional pain medicine with regional anesthesia and nerve blocks. Today, imaging can be combined with interventional techniques for more precise localization of nerves for diagnosis and treatment. The role of artificial intelligence in interventional pain medicine, especially in imaging for interventional procedures, remains unknown but will likely become extremely beneficial.

## Introduction and background

Pain is one of the oldest medical complaints and one about which many questions remain unanswered. While physicians have struggled to understand and manage pain for centuries, the past 40 years have seen a dramatic increase in pain studies, scientific breakthroughs in analgesia, new techniques, and a broad acceptance of the multidisciplinary nature of complete pain therapy [1]. Interventional pain management dates back to the first neural blockade and regional analgesia introduced in 1884. Today, our approach to pain medicine is guided by precise diagnosis and high-technology treatments [2]. These techniques are being employed in greater numbers year after year, often by non-pain specialists. Looking forward to the future in pain medicine, it is of interest to look backward and see how we have bridged the gap from our limited and mainly pharmacologic approaches to pain toward sophisticated image-guided techniques using sophisticated devices and instruments.

The purpose of this article is to provide a summary of the historical milestones in pain medicine, from the original use of opioids as an analgesic to modern uses of ultrasound-guided advanced techniques and possible future directions. This is a historical review based on the authors’ viewpoints as to the most salient developments and key milestones.

## Review

When pain medicine was synonymous with opioids

The “milk of the poppy” was known as an analgesic as far back as 4000 B.C. by the Sumerians in Mesopotamia [3]. While the Sumerians isolated opium from poppies as a pain reliever, it may have first emerged as a drug to produce euphoria rather than analgesia. Arab traders brought opium to China and India around 700 A.D., and by 1200 A.D., opium was already in commerce throughout Europe and Asia Minor [3]. The noted sixteenth-century Swiss alchemist Theophrastus Bombastus von Hohenheim, better known as Paracelsus, introduced laudanum, a tincture of opium made from poppy flower extracts that contained all of the alkaloids found in the poppy plant. Laudanum was recommended for pain control [4]. The earliest reports of tolerance and addiction emerged during this time. In the eighteenth century, Great Britain introduced opium to China, where its use and even production accelerated rapidly. Recreational use of opium was reported in China, as were instances of addiction, to the point that the drug was outlawed except for medicinal use. The Opium Wars of 1839-1842 between Britain and China were a successful effort by Britain to compel China to buy opium from Britain. A subsequent war from 1857 to 1859 involved France, Britain, and the United States against China and opened China up to extensive foreign trade in “unequal treaties,” which put China at an economic disadvantage [5].

Meanwhile, in 1806, German pharmacist, Friedrich Sertürner, isolated a compound in opium, which he named “principum somniferum,” and was marketed in Germany and the United States under the trade name Morphine. Morphine was a name derived from Morpheus, the god of sleep [6]. To this day, morphine remains the single most widely used analgesic medication on earth and appears on the list of the World Health Organization’s essential medicines [7]. Sertürner’s work with morphine established a new pharmacological class of medications, the alkaloids, and created a pain medicine that allowed for controlled dosing [8]. Pierre-Jean Robiquet isolated codeine in 1832 and many other natural substances, including caffeine [9].

In 1856, the introduction of the hypodermic syringe accelerated the use of morphine, which was not regulated and could be purchased in drug stores without a prescription. In 1888, about 15% of all prescriptions dispensed in the city of Boston were for opioids, which were recommended for everything from menstrual cramps to “nervous diseases” and as an antitussive. With this general enthusiasm for the miracle drug morphine quickly came case reports of rampant addiction [10].

**Figure 1:**
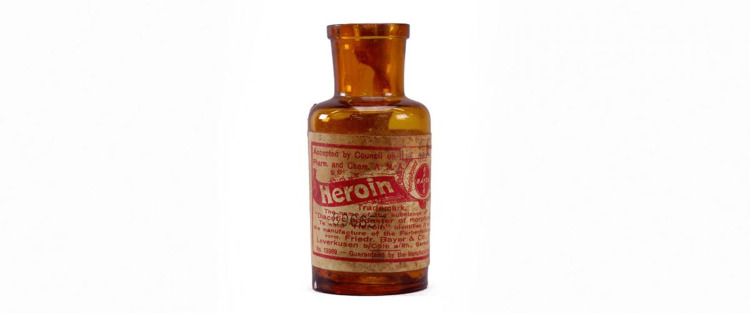
Heroin was originally marketed by Bayer & Co. as a pain reliever less addictive than morphine and as an antitussive. It was synthesized by Felix Hoffmann of Bayer; Hoffmann later created aspirin. Photograph courtesy of the Drug Enforcement Administration Museum in Arlington, Virginia.

In 1883, Heinrich Dreser was working for Bayer, and by acetylating morphine, he created diacetylmorphine. Using the trade name Heroin, this agent was marketed as a “non-addicting formulation of morphine,” a marketing claim that was quickly debunked [11]. Heroin was sold over-the-counter and marketed for pain control and as a pediatric antitussive [12] (Figure 1).

Opioids quickly became drugs of choice to manage pain, induce sleep, improve mood, and manage mental health conditions. This led to their widespread use, proliferation of side effects, morbidity, and potentially life-threatening respiratory depression. As a backlash against the disordered and dangerous use of opioids, a fear and stigmatization of opioids, or “opiophobia,” developed [13]. The Harrison Narcotic Act, passed in 1914, made opium and related drugs the first illegal substances in the United States [14]. While originally intended to better regulate opioids, the Harrison Act was enforced to the extent that it became a prohibition. The backlash against this draconian step occurred in 2004 when the World Health Organization declared access to pain relievers a human right [15], and a few years later, the Declaration of Montreal declared pain control a fundamental human right [16]. These transitions forced a re-examination of opiophobia, but more importantly, they involved a re-evaluation of pain itself.

A physician named John Joseph Bonica (1914-1994) had been a wrestler as a young man, and in adulthood, he suffered from debilitating chronic pain that was not associated with a specific injury or localized tissue damage but rather arose without peripheral associations. Bonica was describing something quite apart from the nociceptive pain triggered by tissue damage or injury with which physicians of that era were familiar. Bonica went on to describe an emotional component to pain and added a psychological dimension that went beyond the physical aspects of pain and pain intensity. Bonica founded the International Association for the Study of Pain (IASP) [17], which recently introduced new pain definitions and recognized that nociplastic pain can occur without observable tissue damage [18]. In 1967, the idea of “constant pain” or unrelenting painful conditions was introduced through the work of a British nurse, Dame Cicely Strode Saunders. Saunders introduced the notion of palliative care and hospice, the idea that comfort, pain relief, and supportive care in a multimodal regimen are sometimes needed for patients for whom curative therapy is not a reasonable goal [19]. Another innovation in pain care occurred around this time with the emergence of multidisciplinary pain clinics that merged medical care with pharmacotherapy, psychological treatments, physiotherapy, and other disciplines to manage pain more holistically. With these transitions, it became imperative that clinicians today treat patients with pain, particularly chronic pain, with empathy and appropriate professional support in the context of the patient’s religious and cultural beliefs.

Pain clinics emerged around this time, bringing a multidisciplinary approach to pain management. This merges psychology, physiotherapy, pharmacology, and other disciplines to better manage pain. It also emerged that, in addition to medical science, drugs, or treatments, it was vital that clinicians have empathy with the patient and provide emotional support in the proper context of that patient’s religious and cultural beliefs. Pain is contextualized within the culture and can color how individuals respond. For example, in some cultures, pain is accepted as a normal part of everyday life; others think it is a sign of good character to bear pain without complaining, while other cultures may encourage people to seek immediate relief from even mild pain. Furthermore, healthcare professionals may also apply their own cultural attitudes and biases toward how they treat patients experiencing pain [20].

The emerging understanding of pain

In 1644, René Descartes discussed phantom pain, which he considered real rather than imaginary, and postulated that pain was experienced in the brain, not in the periphery [21]. Understanding how the brain interprets pain is ongoing work, but the IASP has issued classifications that help clinicians sort through pain. The etiology of pain, in broadest terms, is binary: cancer pain or noncancer pain. The pathophysiology may be nociceptive, which includes somatic and visceral forms of pain; neuropathic, which can be peripheral or central; and the newest category, nociplastic pain, which involves maladaptive aberrant nerve signaling. Pain can also be described by its site or location on the body, by its characteristics (sharp, stabbing, dull, “electric,” migrating), intensity, and duration [22]. One patient may have multiple pain sites with different characteristics, and even a single pain site may be subject to multiple pain mechanisms.

Over a quarter century ago, the World Health Organization (WHO) introduced its now well-known “pain ladder” for the treatment of cancer pain in adults. The concept was that pain should be addressed based solely on its intensity, and treatment should ramp up from weakest to strongest therapeutic options in a stepwise progression [23]. At step one, physicians could prescribe paracetamol or nonsteroidal anti-inflammatory drugs; if pain worsened, weak opioids and combination products such as paracetamol plus codeine were recommended. Strong opioids were prescribed next as pain intensified. Interventional procedures would be the final step. This ground-breaking pain ladder for cancer pain remains in discussion today, although there are certain shortfalls. For one thing, it measures pain in intensity only, and pain treatment may vary depending on the type of pain and whether it is acute or chronic. Second, pain upon first presentation may be very severe and require an opioid analgesic; the idea that severe pain could only be treated with an opioid after nonopioid analgesics were trialed and failed creates unnecessary suffering for patients. Furthermore, the ladder went in only one direction, and sometimes pain, for example, postoperative pain, might require a strong opioid at first and then nonopioid analgesics as pain subsided with tissue healing. In other words, sometimes people might need to go down the ladder and not up. Modifications to the pain ladder have been proposed, and its extension to noncancer pain is recommended [24,25]. The WHO pain ladder opened broader and more confident use of opioid analgesics to treat cancer pain.

Pain physiology requires consideration of the types of nerve fibers that carry pain to the brain. The primary afferent axons were defined by diameter in µm and velocity in meters per second. The A fibers could be divided into α, β, and δ fibers. The α fibers were involved in somatic motor and proprio-nociception; β fibers in touch and pressure; and δ in “fast” pain signaling, the sense of cold, and touch. B fibers, on the other hand, were preganglionic sympathetic in nature. The C fibers are very small-diameter fibers. When found in the dorsal root, C fibers were associated with slow pain transmission and a sense of heat. Sympathetic system C fibers were postgangliotic. A and B fibers are myelinated, but C fibers are not. The fastest and largest-diameter fibers are A, while the slowest and smallest-diameter fibers are C, with B in the middle. 

The body has its own system to modulate pain, but these mechanisms are not fully elucidated. The Gate Control Theory maintains that there can be segmental inhibition of pain in that only a certain number of pain signals can be carried at one time before they block each other out [26]. The body also has an endogenous opioid system that delivers enkephalins, endorphins, and dynorphins to help regulate pain. A descending inhibitory nervous system, modulated in part by norepinephrine and serotonin, controls what types of nociceptive information may travel to the brain from the periphery [27].

In 1965, Ronald Melzack and Patrick D. Wales proposed the Gate Control Theory of Pain, which theorized that within the spinal cord there was a mechanism that determined which pain signals from the periphery could be transmitted to the brain for processing; in other words, there was a “gate” that allowed certain signals to come through while blocking others [28]. The Gate Control Theory described that the Aβ nerve fibers can close the gate by activating potentially inhibitory interneurons but the gate can open when inputs from C fibers deactivate the inhibitory interneurons and the Aδ fibers take over as primary afferents to activate the neurons for spinal cord transmission. The mechanisms underlying these actions remain to be elucidated, but various glutamate receptors and electrical filtering of the potassium channels performed by dendrites have been implicated [29].

Prostanoids are involved in the inflammatory response, chronic inflammation, and painful symptoms, and the inhibition of the cyclo-oxygenase (COX) enzymes can reduce prostanoid and prostaglandin production, reducing both pain and inflammation [30]. Among the many nonsteroidal anti-inflammatory drugs that inhibit prostaglandin synthesis are ibuprofen, ketoprofen, diclofenac, naproxen, piroxicam, meloxicam, and others.

Pain may also be treated using adjuvant agents, such as the anticonvulsants carbamazepine, gabapentin, and pregabalin, or sodium-channel blockers, such as ketamine or calcium-channel blockers, such as ziconotide. These are not opioids but may be used as monotherapy or combined with other analgesics (Table 1).

**Table 1:** Types of opioids. Note that diacetyl morphine, or heroin, is a prescription drug in Canada and some European nations, but it is not legal in Latin America or the United States [31].

Alkaloid opioids	Semi-synthetic opioids	Synthetic opioids	Peptide Opioids
Morphine, Codeine, Thebaine, Noscapine, Papaverine	Hydromorphone, Oxycodone, Heroin (diacetyl morphine), Etorphine. Antagonists: Naloxone, Naltrexone	Nalbuphine, Levoranol. Butorfanol, Pentazocine, Methadone, Tramadol, Meperidine, Fentanyl, Alfentanil, Sufentanil. Remifentanyl	Endorphin, Enkephalin, Dynorphin

While one might think that problems related to opioid dependence and opioid use disorder are problems relegated to the developed world, it is important to note that tramadol use disorder has emerged as a serious problem in sub-Saharan Africa [32]. Tramadol can be purchased over-the-counter in many parts of the world, including Africa. As the use of opioids in these nations rises, so does the number of opioid-related deaths; this very much follows the experience of the United States. Problematic and nonmedical use of tramadol has been reported in Egypt [33], Ghana [34], South Africa [35], and other African nations [36].

In Mexico, President Andres Manuel Lopez Obrador proposed a ban that would prohibit the use of fentanyl even in surgical or medical settings [37]. While not yet approved, such a prohibition places serious constraints on anesthesiologists, whose patients require strong opioids during operations. This proposal would be medically dangerous if made law [38].

Opioids are underutilized in Latin America, although scientific evidence supports that opioids are medically necessary, safe, and effective analgesics when used in appropriate patients under clinical supervision [38,39]. Inappropriate and inadequate pain control is a serious public health issue on the one hand, but indiscriminate over-prescribing of opioids is associated with morbidity and mortality on the other hand. Latin America needs public policies, regulation, and educational efforts for the proper use and monitoring of opioid analgesics.

How the hollow needle changed pain treatment

**Figure 2:**
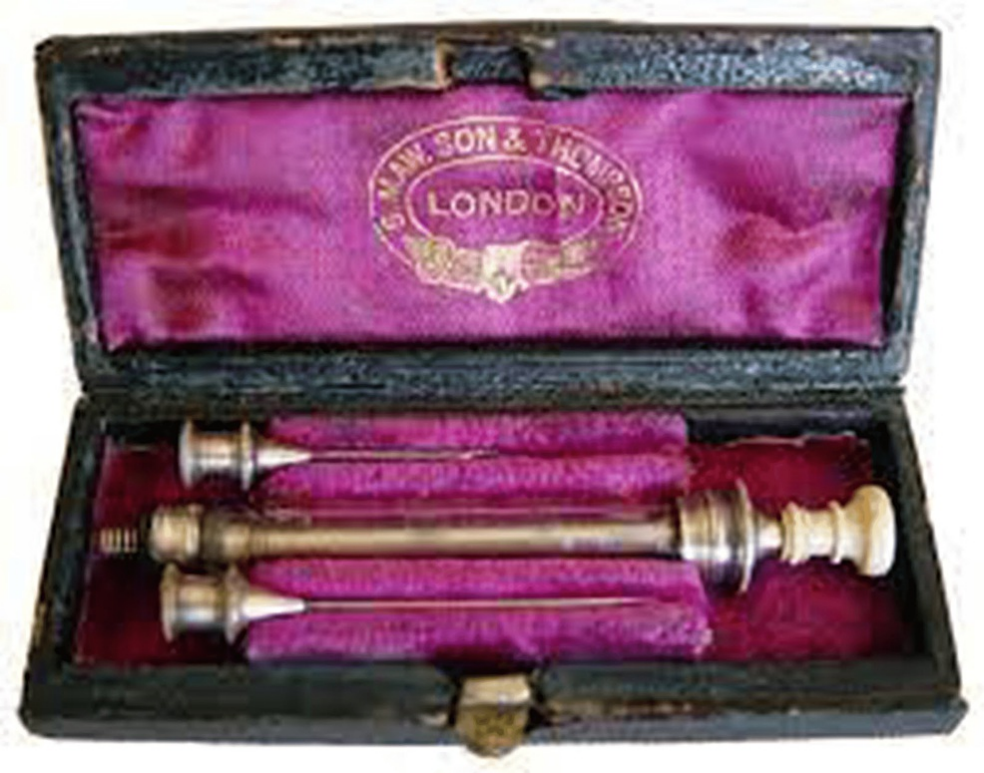
An example of an original Pravaz metal syringe with narrow-tipped hollow needle in its original velvet-lined case. Novel with this syringe was the transparent barrel and the screw at the proximal end of the syringe, which could be manually adjusted for control of the dose. This photo by unknown artist is licensed under Creative Commons.

In 1844, Francis Rynd, an Irish physician (1811-1861), developed a hollow needle without a sharp point that could be used for subdermal fluid infusions. In 1853, a physician in Scotland seeking to treat his wife for pain as she was dying of terminal cancer used this hollow needle and a syringe to create the first hypodermic needle with which he could inject her with morphine for analgesia. Alexander Wood was the first physician to treat pain in that manner, and his dying wife was the first person to be administered morphine injections for pain control. Wood used the device to deliver medicine subcutaneously rather than intravenously. Charles Pravaz in France developed the first practical metal syringe for use with a fine-tipped, sharp hollow needle. The first use of these syringes was for intra-auricular injections, but they were soon used to inject opioid analgesics as well. This invention, born of necessity, changed clinical practice forever. In fact, Alexander Wood obtained a patent for the use of his syringe to inject morphine for pain and even marketed it as a “narcotic injection syringe” [40]. A surgeon in London named Charles Hunter was the first to call this a hypodermic syringe. The syringe was later simplified by William Ferguson, which made it easier and more practical to manufacture. An instrument maker in France known as Luer developed the conical nozzle known by his name (Figure 2). Single-use syringes were first introduced in the 1960s [40].

For surgical interventions, non-steroidal anti-inflammatory drugs (NSAIDs) and steroids were needed to blunt the inflammatory response, while opioids were required to increase descending inhibition, and lidocaine blocked sodium channels. This concept of “balanced analgesia,” first introduced into the literature by John S. Lundy in 1926, allowed for different agents with different mechanisms of action to produce the variety of different effects needed for anesthesia [41]. This is differentiated from multimodal analgesia, introduced in 1993, which combined balanced analgesia with analgesic techniques such as nerve blocks [41]. The Meyer-Overton correlation found that increasing the lipid solubility of an agent by increasing its chain length resulted in an increase in anesthetic potency [42]. Meyer and Overton worked separately in Europe, the former in Switzerland and the latter in Germany, in the late nineteenth century and identified the lipid membrane as the site of the anesthetic action of pharmaceutical products. Their groundbreaking work has been challenged, but not overthrown, with new work on the putative role of proteins in anesthesia [42].

The advent of regional anesthesia and nerve blocks may be traced back to a report from 1884 by a physician named Carl Koller, a colleague of Sigmund Freud, both of whom had an interest in the medical use of cocaine. Koller found that cocaine was an excellent local anesthetic, and it was used in a variety of procedures, including ophthalmologic procedures, urological operations, and general surgery [43]. In the late nineteenth century, Dr. J. Leonard Corning used a 3% cocaine solution to perform lumbar punctures, first in dogs and then between T11 and T12 in a male patient, marking the first peridural blockade. At almost the same time, a surgeon named August Bier used a cocaine injection into the subarachnoid space of the spine for what is considered the first report of spinal anesthesia [44]. Regional blocks were first described in 1901 when radiologist J.A. Sicard injected diluted cocaine into the sacrum of a patient to help reduce low back pain [45]. Around this time, regional blockades were used for surgery as well as pain control.

These techniques gave rise to what has become known as interventional pain management, a specialty field that has had numerous innovations in pain care [1]. Interventional pain medicine is a discipline dedicated to the diagnosis and treatment of pathologies related to pain, mainly by using interventional techniques to help control chronic, persistent, and refractory pain in a way that is independent from or related to other modalities of treatment [46].

Percutaneous injections were made possible by the hollow needle, expanding the role of regional anesthesia in surgical procedures as well as for pain management [47]. For example, neurolytic superior hypogastric plexus block was shown in a study to provide safe and effective pain relief in patients with visceral pain while, at the same time, cutting their opioid use by 43% [48]. Cervical epidural opioid administration was reported to be effective against pain in a pediatric case study [49]. Using a hollow needle, acrylic cement can be used to treat certain types of vertebral angiomas that may be unsuitable for radiotherapy or other treatments [50]. In 2003, Plancarte and colleagues described a transdiscal approach for splanchnic nerve inhibition for control of visceral pain [51]. The hollow needle allows for the delivery of pharmacologic products, fiberoptics for diagnosis, catheters, and radiofrequency (RF) ablation tools. The hollow needle supports many types of devices and treatments, including a variety of imaging techniques.

In fact, it is the combination of the hollow needle for delivery of drugs or tools with imaging, such as fluoroscopy, that has opened even more opportunities by allowing the physicians to view in real time the precise location and appropriate angling of the needle and to monitor the delivery of medication or energy.

In terms of the future, interventional medicine may be able to help solve the problem of osteogenic pain by giving physicians better tools to study unique bone structures and to deploy substances such as cement for repair, agents for neurolysis, and radiofrequency energy. Under fluoroscopic guidance, the hollow needle in the hands of skilled interventional pain physicians can use multiple devices and techniques to conquer pain. More and more interventional procedures are carried out every year because they are safe and effective. But as interventional procedures become more familiar and widespread, they may be used indiscriminately, leading to inappropriate patient selection, disregard of evidence-based findings in the literature, and poor adherence to guidelines. Optimal results with interventional pain medicine require not only innovation and research but also investment in the training and education of physicians and the health literacy of our patients [52].

From blind techniques to ultrasound and beyond

Today, ultrasound (US) imaging is widely recognized for its ability to help guide interventional procedures, offering real-time images in multiple planes using a relatively simple technique with no radiation. The use of the US can benefit static or dynamic examinations. The three main fields for US guidance in interventional medicine are neuraxial injections (such as lumbar spine injections), nerve management (such as peripheral nerve blocks and nerve decompression), and musculoskeletal pain management [53].

However, until the turn of the millennium, the benefits of ultrasound imaging were not well recognized. In 1953, Daniel Charles Moore (1918-2015) wrote about a variety of regional anesthetic techniques with the slogan, “no paresthesia, no anesthesia!” The idea was that paresthesia served to confirm the placement of the needle’s tip at the epineurium of the nerve, which, in turn, offered a strong probability of adequate analgesia [54].

Raj P. Prithvi (1932-2016) was a pioneer of regional anesthesia who used a blind technique with nerve stimulation to identify and localize various nerves [55]. He was a colleague of Alon Palm Winne (1932-2015) and developed a similar blind technique for a single-injection brachial plexus block that likewise relied on anatomic landmarks for guidance rather than imaging [56]. The use of electrical nerve stimulators for localizing peripheral nerves for nerve blocks was recommended. Indeed, the electrolocalization of nerves gained popularity in the 1990s. This led to a challenge to the model of seeking paresthesia for confirmation; in fact, it was proposed that trying to achieve paresthesia during a peripheral nerve block might cause neurological damage, although they conceded that a “gentle touch” with a needle would not likely damage a normal nerve [57].

Moore argued that his model of seeking paresthesias applied to certain specific types of peripheral nerve blocks, such as the brachial plexus or sciatic nerves, among others [54]. The question is not entirely resolved in the minds of pain specialists even today. The evidence in the literature does not unequivocally support one technique over the other with respect to adequate nerve blockade or the rates of postoperative neurologic damage, but some physicians may prefer one method over the other [58].

In 1994, Jerry Vloka and Admir Hadzic, two physicians from the New York School of Anesthesia, founded NYSORA, an organization dedicated to the promotion of regional anesthesia, pain management, and perioperative medicine by means of education initiatives [59]. It was partly through the leading-edge innovations of NYSORA that nerve stimulators were combined with US guidance. While the transition from paresthesia to a nerve stimulator for confirming needle placement was a difficult transition for many interventional pain physicians, the leap from the use of anatomical landmarks alone to an US-guided method was far more manageable.

US guidance offers numerous advantages to the interventional pain physician. It is a safe, low-cost, readily accessible, and noninvasive system that is widely used and understood. US machinery is portable, and cost-effective, and generally available in most institutions and clinics. A major advantage of US guidance is that it can be used in real time to guide procedures. Perhaps most importantly, there are no radiation risks at all with this imaging technique, meaning it can be used as needed without concerns about exposing the patient to unsafe levels of radiation. There are certain limitations with US systems in that their use can be dependent on the operator’s expertise, and there is a steep learning curve to learn how to use them for the best results. US devices may produce artifacts that can impede easy interpretation. There is also a problem with resolution X-depth, meaning that at deep tissue levels, images can lose resolution. For interventionalists, this can make spinal imaging particularly challenging. A novel approach to managing this imaging problem is to fuse magnetic resonance imaging (MRI) and/or computed tomography (CT) scans with US images. Technology exists to create fused images that allow for easy interpretation as anatomic structures such as bones become readily recognizable [60].

While still in its infancy, artificial intelligence (AI) may greatly improve our imaging abilities and our ability to use US images. For example, AI is already used with some breast US images to recognize and mark specific anatomical structures [61]. No doubt AI will have imaging applications for musculoskeletal pain, low back pain, regional anesthesia, and other procedures related to anesthesia and analgesia [62].

## Conclusions

Pain diagnosis and treatment must be one of the oldest fields of medicine, but it is continually improving and advancing. The hollow needle, percutaneous drug delivery, regional anesthesia, and nerve blocks offer safe and effective forms of anesthesia and analgesia for patients. The use of US imaging techniques offers more precise control for nerve localization, and future fused images can provide good insight into the difficult-to-image structures of the spine. Osteogenic pain is an important new area for interventional techniques since there are few other options for patients with bone disorders. The future of pain medicine is still being written, with the next chapter likely to be how artificial intelligence is incorporated into interventional pain medicine, particularly in terms of its role in imaging and identification of particular bony structures. 
